# Evolution of Surface Nanopores in Pressurised Gyrospun Polymeric Microfibers

**DOI:** 10.3390/polym9100508

**Published:** 2017-10-13

**Authors:** U. Eranka Illangakoon, Suntharavathanan Mahalingam, Rupy K. Matharu, Mohan Edirisinghe

**Affiliations:** Department of Mechanical Engineering, University College London, London WC1E 7JE, UK;suntharavathanan.mahalingam@ucl.ac.uk (S.M.); rupy.matharu.15@ucl.ac.uk (R.K.M.); m.edirisinghe@ucl.ac.uk (M.E.)

**Keywords:** pressure, gyration, polymer, microfibers, nanopores, surface

## Abstract

The selection of a solvent or solvent system and the ensuing polymer–solvent interactions are crucial factors affecting the preparation of fibers with multiple morphologies. A range of poly(methylmethacrylate) fibers were prepared by pressurised gyration using acetone, chloroform, *N,N*-dimethylformamide (DMF), ethyl acetate and dichloromethane as solvents. It was found that microscale fibers with surface nanopores were formed when using chloroform, ethyl acetate and dichloromethane and poreless fibers were formed when using acetone and DMF as the solvent. These observations are explained on the basis of the physical properties of the solvents and mechanisms of pore formation. The formation of porous fibers is caused by many solvent properties such as volatility, solubility parameters, vapour pressure and surface tension. Cross-sectional images show that the nanopores are only on the surface of the fibers and they were not inter-connected. Further, the results show that fibers with desired nanopores (40–400 nm) can be prepared by carefully selecting the solvent and applied pressure in the gyration process.

## 1. Introduction 

Ultra-thin fibers have been generated by using various fabricating techniques such as self-assembly, phase separation, drawing, template synthesis, electrospinning [1,2,3,4] and pressurised gyration [5,6,7,8]. Among these state-of-the-art techniques, more recently discovered pressurised gyration has attracted much attention due to higher production rate and low cost. Since 2013, this technique is widely used to prepare nano- to micro-scale fibers for various applications such as drug delivery [9] and biopharmaceutical applications [8,10]. The pressurised gyration method utilises both centrifugal spinning and solution blowing simultaneously [5].

The basic gyration set-up consists of a rotary aluminum vessel, which contains a series of pin-hole type orifices along its circumference, a high-pressure gas supply, a DC motor and a speed controller. The top of the rotary vessel is connected to a high-pressure gas supply that is capable of producing pressures up to 3 × 10^5^ Pa, and the bottom is connected to a DC motor—which is used to rotate the vessel. The polymer solution is placed in the gyration vessel. The centrifugal force created due to high-speed rotation together with the high-pressure gas supply consequently results in fibers extruding through the orifices of the gyration vessel. The polymer jet travels through air during which the solvent evaporates and solid fibers are deposited on the collector. Previous work on pressurised gyration shows that fiber diameter and morphology can be changed by varying the polymer concentration, rotational speed of the vessel and the working pressure [5,7,11]. Recent studies of polymer-protein solutions have also shown that pressurised gyration is capable of generating microbubbles rather than fibers by controlling the rotational speed and applied pressure within a certain range [9].

Polymer–solvent interactions determine the properties of the spinning solution [12]. When a polymer is highly soluble in a solvent, it will form strong polymer–solvent interactions where the polymer chains swell and expand to maximise the intermolecular interactions and when a polymer is less soluble the polymer chains contract and stay closer to each other to minimise the polymer–solvent interactions [6,13]. It is well known that the spinning solution parameters strongly depend on the polymer and solvent used to dissolve the polymer. Therefore, the properties of the solvent such as boiling point, surface tension, vapour pressure and the solution parameters show a profound effect on fiber morphology. The effect of solvents on electrospun nanofibers were reported by various researchers using different polymers such as poly(methylmethacrylate) (PMMA) [14,15,16], zein [17], polystyrene [18], poly(ε-caprolactone) [4,16] and cellulose acetate [19]. These reports have showed that fibers with hierarchical structures can be obtained when using solvents with higher vapour pressure [20]. It is a well-known phenomenon that a volatile solvent plays a pivotal role in the generation of fibers with different surface morphologies such as porous, wrinkled and smooth [1,14,21]. Even though the formation of these hierarchical structures is not fully understood, phase separation and breath figure approach can be used to explain the formation of these structures. Fibers with hierarchical secondary structures have advantages compared to fibers with smooth surfaces such as higher surface-area-to-volume ratio, super-hydrophobicity or super-hydrophilicity, high rate of adsorption, low density and high surface volume and fibers with these characteristics can be used in various areas such as drug delivery, tissue engineering and electronics [22,23,24].

PMMA is an amorphous, transparent thermoplastic polymer widely used in biomedical applications such as bone implants [25,26], prosthetics [27], dentistry [28,29,30], drug delivery [31], cosmetic surgeries and as intraocular lenses [32] implanted after cataract surgery. PMMA was selected as the model polymer for this study due to its high solubility in a wide range of solvents. Hierarchical structures of PMMA were prepared by several researchers [33,34] using electrospinning as the fiber-making technique. However, such a study has not been performed using pressurised gyration and, in this work, we are reporting the formation of hierarchical structures of PMMA using this forming route.

## 2. Experimental Details

### 2.1. Materials

Poly(methylmethacrylate) of molecular weight 120,000 g/mol, chloroform, acetone, *N,N*-dimethylformamide (DMF), ethyl acetate and dichloromethane (DCM) were obtained from Sigma-Aldrich (Gillingham, UK). All reagents were analytical grade and were used as received.

### 2.2. Preparation of Spinning Solutions

A series of 20 wt % PMMA solutions were prepared by dissolving PMMA in chloroform, acetone, *N,N*-dimethylformamide (DMF), ethyl acetate and dichloromethane (DCM). All the polymer solutions were magnetically stirred for 24 h to obtain homogeneous solutions.

### 2.3. Pressurised Gyration 

**Figure 1:**
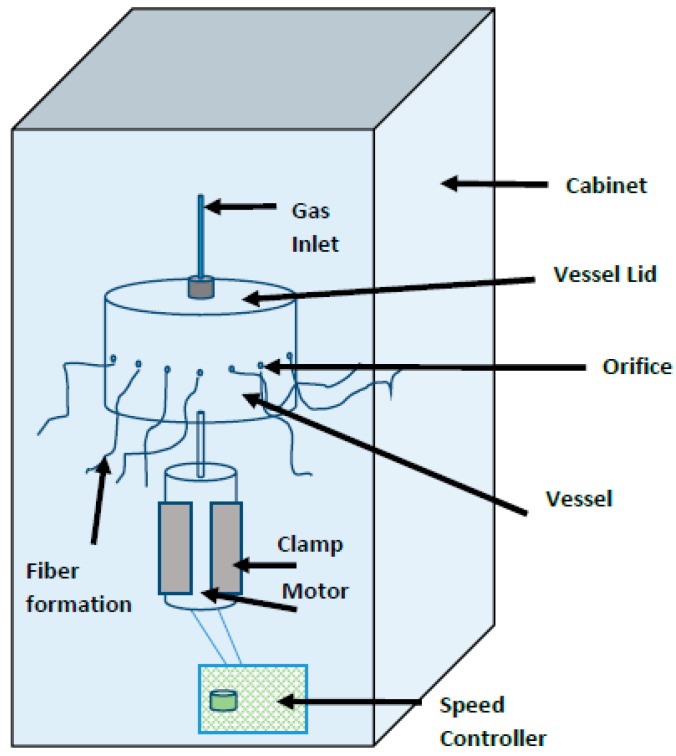
Experimental set-up of the pressurised gyration rig used in this work.

Figure 1 shows the experimental set-up of the pressurised gyration process. The rotary aluminum cylindrical vessel (∼60 mm in diameter and ∼35 mm in height) contains 24 orifices on its face, each having a diameter of 0.5 mm; 6 mL of the polymer solution was placed in the vessel and spun at 36,000 rpm using 0.1 MPa working pressure. Polymer solutions made using chloroform, DCM and ethyl acetate were spun at 0, 0.2, 0.3 MPa to obtain fiber samples under different working pressures. PMMA fibers were collected using a rod-collector placed 100 mm away from the vessel. All the spinning experiments were carried out under ambient conditions (25 ± 2 °C and relative humidity 47% ± 3%).

### 2.4. Fiber Characterisation

#### Scanning Electron Microscopy (SEM)

The fiber morphology was assessed using SEM (Quanta 200 FEG ESEM, FEI, Hillsborough, OR, USA and JEOL JSM-6301F, Peabody, MA, USA). Prior to imaging, the samples were coated with 20 nm of gold under argon using a Quorum Q150T Turbo-Pumped Sputter Coater. All SEM images were captured at an acceleration voltage of 5 kV. The average fiber diameter and average pore diameter was determined by measuring diameters and pores at over 50 points in the SEM images using the ImageJ software (National Institute of Health, Bethesda, MD, USA). 

## 3. Results and Discussion

### Evaluation of Microstructure of PMMA Fibers

The selection of an appropriate solvent or solvent system to prepare polymer solutions based on the solubility parameters between the polymer and solvent/solvent system is a crucial step in any fiber forming process. The solvent with the highest solubility is not always the best solvent for the production of fibers as the solvents physical parameters, such as surface tension, viscosity, vapour pressure and boiling point affect the spinning process [18]. The Flory–Huggins interaction parameter- chi(χ_12_) [35] gives a measure of the interaction of the polymer chains with the solvent molecules as well as the polymer–polymer interaction. The χ_12_ parameter for polymer solutions was calculated from the equation given below [36].

χ_12_ = (δ_p_ − δ_s_)^2^ × (*V*_mol_/R*T*) (1)

In here, subscripts s and p denote solvent and polymer, respectively. δ and *V*_mol_ are the solubility parameter and the molecular volume, respectively, of the solvent at temperature *T* (298 K). R is a universal gas constant. In general, solvents with χ_12_ between 0 and 0.5 are considered as good solvents and the solvents with χ_12_ > 0.5 are considered as poor solvents [37]. The calculated χ_12_ values for each solvent are listed in Table 1. It is found that χ_12_ for acetone, chloroform, DCM and DMF is <0.5, hence these can be classified as good solvents for PMMA. In these solvents, PMMA chains overlap less and there is a greater interaction with the polymer–solvent molecules. The above equation does not account for secondary forces such as polar/non-polar interaction and hydrogen bonding. Therefore, there are exceptions; the calculated χ_12_ for ethyl acetate is 0.64, even though ethyl acetate has this high χ_12_ value it was able to dissolve PMMA due to its high polarity and greater ability to make hydrogen bonds with PMMA chains.

Furthermore, the degree of affinities between polymer and solvent can be expressed by the solubility parameter distance *R*_a_. Values of *R*_a_ for the solvents used in this study were calculated using Hansen solubility parameters for each solvent using Equation (2) [38] and these are listed in Table 1.

*R*_a_ = [4(δd_2_ − δd_1_)^2^ + (δp_2_ − δp_1_) ^2^ + (δh_2_ − δh_1_)^2^]^1/2^ (2)

Here, δd, δp, and δh are the solubility parameters representing the dispersion, polar and hydrogen bonding contributions, respectively. Subscripts 1 and 2 represent the solubility parameters for polymer and solvent, respectively. The calculated values *R*_a_ show that Acetone (*R*_a_ = 3.72) and DMF (*R*_a_ = 3.99) show higher affinity to PMMA compare to other solvents. This may be one of the factors that cause non-porous fibers with these solvents.

**Figure 2:**
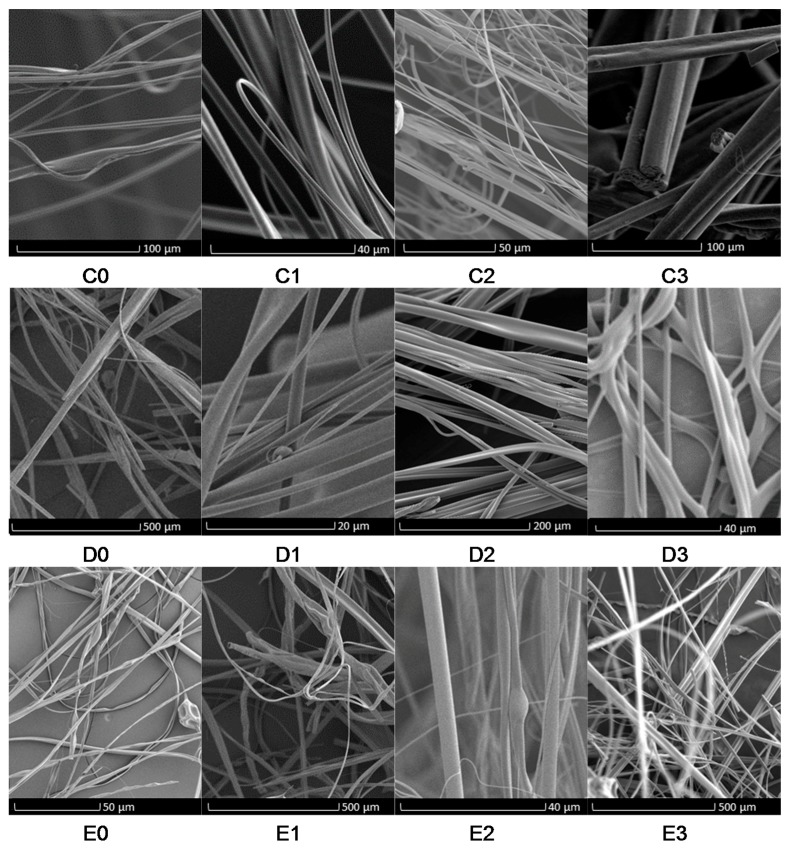
Scanning electron micrographs of poly(methylmethacrylate) (PMMA) fibers prepared using different solvents and different working pressure: (**C0**–**C3**) fibers made using chloroform 0, 0.1, 0.2, 0.3 MPa working pressure, respectively; (**D0**–**D3**) fibers made using DCM 0, 0.1, 0.2, 0.3 MPa working pressure, respectively; (**E0**–**E3**) fibers made using ethyl acetate 0, 0.1, 0.2, 0.3 MPa working pressure, respectively.

**Figure 3:**
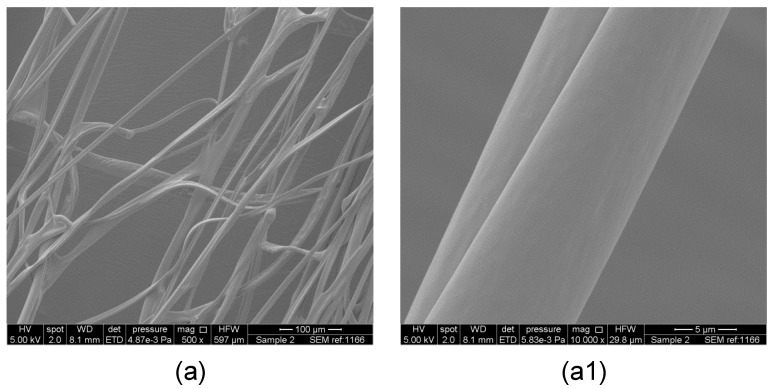

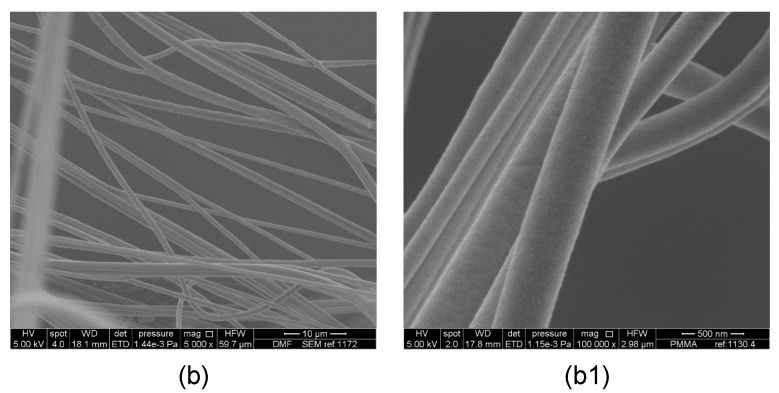
Scanning electron micrographs of PMMA fibers prepared using (**a**,**a1**) acetone as the solvent and (**b**,**b1**) *N,N*-dimethylformamide (DMF) as the solvent.

**Figure 4:**
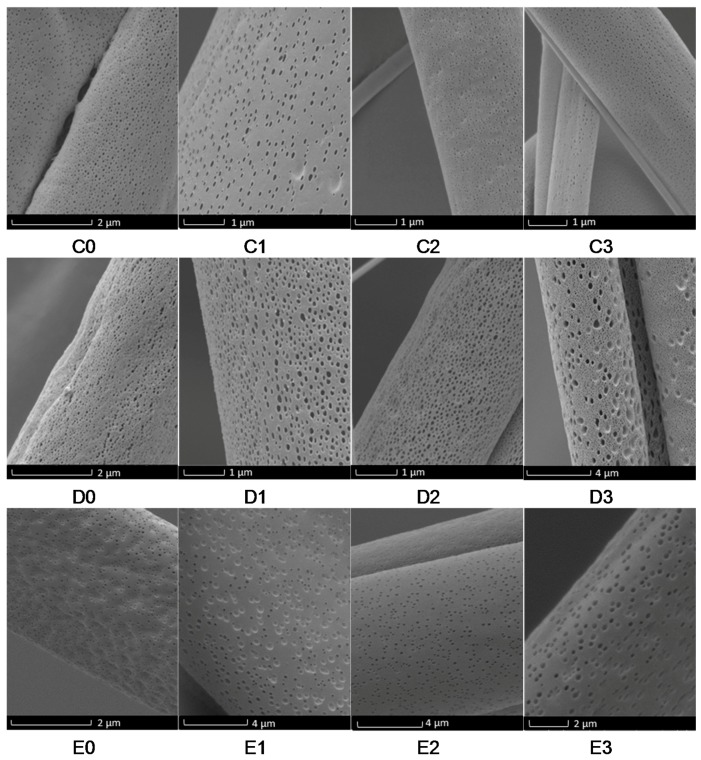
Higher magnification scanning electron micrographs of PMMA fibers prepared using different solvents and different working pressure: (**C0**–**C3**) fibers made using chloroform 0, 0.1, 0.2, 0.3 MPa working pressure, respectively; (**D0**–**D3**) fibers made using DCM 0, 0.1, 0.2, 0.3 MPa working pressure, respectively; (**E0**–**E3**) fibers made using ethyl acetate 0, 0.1, 0.2, 0.3 MPa working pressure, respectively.

**Figure 5:**
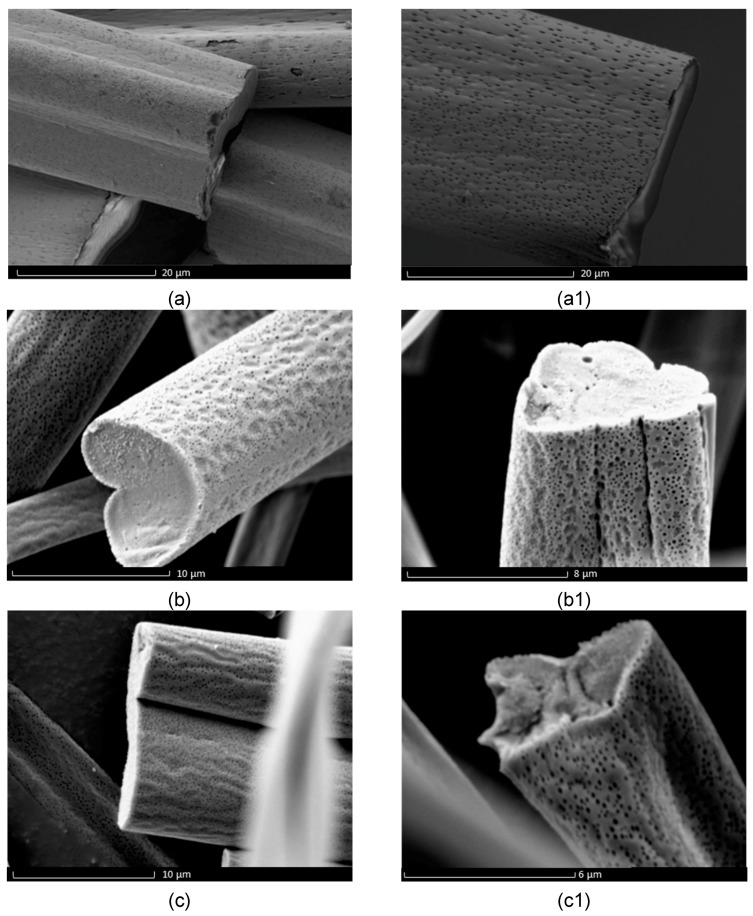
Scanning electron microscope images of cross sections of PMMA fibers: chloroform (**a**,**a1**); dichloromethane (DCM) (**b**,**b1**); ethyl acetate (**c**,**c1**).

Figure 2, Figure 3 and Figure 4 show the low- and high-magnification images of the fibers obtained from various solvents. Samples prepared from chloroform, DCM and ethyl acetate exhibit surface pores while samples from acetone and DMF did not show any surface pores. The cross-sectional images of the samples are shown in Figure 5 and indicate that the pores are only a surface feature. In previous literature, the formation of hierarchical structures from different solvents was mainly attributed to the volatility of solvent, phase separation and due to breath figures [1,4]. These researchers paid less attention to the solubility of polymer in each solvent. The formation of breath figures is a very complicated process and there is no common mechanism to explain all experimental results [39]. When highly volatile solvents such as chloroform, ethyl acetate and DCM start to evaporate, the temperature at the air–liquid interface will decrease rapidly due to the enthalpy of vaporization. It was reported that the temperature of a chloroform solution can fall between 0 to −6 °C during evaporation [40]. This temperature drop significantly lowers the dew point of the atmosphere; thus, water vapour can condense on the surface of the fiber at random positions. These water droplets then sink in to the fiber core or cause indentations on the surfaces. When water droplets evaporate from the fibers, their imprints remain as pores on the fibers [41,42]. Pores are only on the surface because chloroform, ethyl acetate and DCM are immiscible with water and this limits the penetration of these water droplets to the fiber core. Also, the rate of solvent evaporation on the surface of the polymer jet and the solvent diffusion from the core of the jet to the surface of the polymer jet determines the final structure of the fiber.

The measured fiber diameters of the fabricated fibers are listed in Table 2. The fiber diameter data show that the smallest microfibers (1 ± 0.4 µm) were obtained when using DMF as solvent and the largest microfibers (11 ± 3 µm) were obtained when using acetone as solvent. Like our previous work with PEO/water system [5], the fiber diameter did not significantly decrease with increase of applied pressure in the PMMA/solvent systems. This is likely to be caused by the highly volatile solvents used in this study. Therefore, increased air flow caused by increase in applied pressure seems not to appreciably increase polymer jet elongation, resulting in fibers with roughly similar diameters. The PMMA fibers obtained from DMF show smooth surfaces (Figure 3). DMF has less volatility compared to other solvents; therefore, DMF evaporation from the surface is slower than the DMF diffusion from the core. This allows the formation of cylindrical fibers. DMF and water vapour are miscible. Therefore, the water vapour formed due to DMF evaporation can diffuse into the polymer jet rather than deposit on the polymer jet, resulting in fibers with a smooth surface. However, it should be noted well that recent work done by Kuroda et al. [43] described the formation of surface pores on a hyperbranched polystyrene film when using tetrahydrofuran as the solvent which is highly miscible with water. If the solvent evaporation on the surface is faster than solvent diffusion from the core, it will result in flat fibers with ribbon-like cross-sections [12]. This was observed when using acetone as the solvent, resulting in fibers with larger diameter. Acetone volatility is relatively similar to chloroform, ethyl acetate and DCM. However, these fibers were not porous (Figure 3). From this observation it can be concluded that formation of porous fibers is not purely based on solvent volatility. When considering acetone–PMMA, DMF–PMMA interaction, χ_12_ values are 0.15 and 0.22, respectively, which are relatively similar to chloroform and DCM. This suggests that formation of pores is not purely due to polymer–solvent interaction. The δ*P* values for acetone (10.4) and DMF (13.7) are significantly high compared to chloroform (3.1), DCM (6.3) and ethyl acetate (5.3). This may be the reason for obtaining fibers with smooth surfaces.

It was observed from SEM images (C0, D0, E0) that fibers prepared without applied pressure (0 MPa) have rough/non-uniform surfaces compared to fibers formed with applied pressure. This is possibly due to the different rate of solvent evaporation in the absence of the working pressure. Further, it was observed that when the applied pressure increases from 0.1 to 0.3 MPa, the fiber diameter was slightly reduced for all three solvents (Table 2). This is because higher working pressure promotes polymer jet elongation thereby reducing fiber diameter. A similar trend was observed with other polymers such as poly(ethylene oxide) [5] and Nylon [44].

The pore diameters of the fabricated fibers are shown in Table 3. A clear trend between the applied pressure and pore diameter cannot be established from the experimental observations. The formation of surface pores is a complex process as described in pervious literature [4,41]. The temperature change on the surface of the polymer jet during solvent evaporation plays a key role in the formation of these nanopores [4]. Therefore, varying the applied pressure during gyration alters the rate of solvent evaporation and, consequently, the temperature of the surface of the fiber jet. However, presented data clearly shows that fibers with desired nanopores (ranging from 40 to 400 nm) can be fabricated by carefully selecting the solvent and applied working pressure. Formation of nanopores and their size cannot be effectively controlled during electrospinning by varying processing parameters such as applied voltage, flow rate or collection distance. This is a unique advantage of our process compared to electrospinning and other porous structure-making techniques.

The nanopores on the fibers formed using chloroform were elliptical in shape and their longer dimension is oriented with the fiber axis, while the pores in fibers from DCM and ethyl acetate were more spherical (Figure 4). Formation of porous electrospun PMMA fibers were previously reported when using chloroform, DCM and ethyl acetate as the solvent [14,16]. These observations cannot directly compare with gyrospun fibers as the surface of the polymer jet in gyration is charge-free compared to the polymer jet in electrospinning. The electrical charge in the electrospun jet slows down solvent evaporation from the jet and thereby retards phase separation [16]. Furthermore, fibers formed using ethyl acetate showed bead-on string morphology. Such structures are formed when the concentration of polymer in solution is either low or high compared to critical spinning concentration [45]. When considering χ**_12_** values, the highest value is obtained for ethyl acetate, which is 0.64, indicating that ethyl acetate interacts less with PMMA, thereby resulting in beaded fibers.

## 4. Conclusions

In this study, we examined the formation of different hierarchical structures of PMMA by varying the solvent. It was clearly evident from the interaction and solubility parameters that each solvent behaves differently with respect to PMMA. The swelling of PMMA chains and their chain engagement can be predicted and shows that polymer–solvent interactions vary from solvent to solvent and these interactions play a major role in formation of the hierarchical structures. This study uncovered that by selecting appropriate solvent and applied pressure, it is possible to fabricate microfibers of PMMA with various surface morphologies using pressurised gyration. The creation of microfibers of PMMA with nanopores on the surface is a distinctive outcome. 

## Figures and Tables

**Table 1:** Physical properties of PMMA and solution parameters of solvents used in this study. δ and χ values were obtained from reference [36].

	PMMA	Chloroform	Acetone	DMF	Ethyl Acetate	DCM
Molecular Volume (cm^3^·mol^−1^)	-	79.70	73.52	76.95	98.50	63.90
Vapour pressure (mm/Hg)	-	160	184	2.70	73	353
Vapour density (vs.·air)	-	4.1	2	2.5	3	2.9
Boiling point (°C)	-	61	56	153	77	40
δD (J·cm^3^)^1/2^	17.00	17.80	15.50	17.40	15.80	18.20
δP (J·cm^3^)^1/2^	5.80	3.10	10.40	13.70	5.30	6.30
δh (J·cm^3^)^1/2^	9.20	5.70	7.00	11.30	7.20	6.10
δ (J·cm^3^)^1/2^	22.20	18.95	19.93	24.86	18.20	20.20
χ_12_	-	0.34	0.15	0.22	0.64	0.10
*R*a	-	8.25	3.72	3.99	5.98	5.67

**Table 2:** Measured fiber diameter of the fabricated fibers.

Pressure (MPa)	Fiber Diameter (µm)				
	Chloroform	Dichloromethane	Ethyl Acetate	DMF	Acetone
0	2.9 ± 2.5	5.5 ± 2.2	4.5 ± 2.3	-	-
0.1	3.3 ± 1.2	4.3 ± 2.1	5.1 ± 1.1	1 ± 0.4	11 ± 3
0.2	2.9 ± 1.5	3.9 ± 1.6	4.8 ± 2.3	-	-
0.3	2.8 ± 1.8	3.7 ± 1.9	4.7 ± 2.8	-	-

**Table 3:** Measured pore size of the fabricated fibers.

Pressure (MPa)	Pore Size (nm)		
	Chloroform	Dichloromethane	Ethyl Acetate
0	54 ± 12	42 ± 12	121 ± 23
0.1	126 ± 18	126 ± 33	199 ± 54
0.2	109 ± 20	104 ± 43	400 ± 80
0.3	44 ± 10	220 ± 180	124 ± 23
